# Spatial correlations between MRI-derived wall shear stress and vessel wall thickness in the carotid bifurcation

**DOI:** 10.1186/s41747-018-0058-1

**Published:** 2018-10-10

**Authors:** Pim van Ooij, Merih Cibis, Ethan M. Rowland, Meike W. Vernooij, Aad van der Lugt, Peter D. Weinberg, Jolanda J. Wentzel, Aart J. Nederveen

**Affiliations:** 1 Department of Radiology & Nuclear Medicine, Amsterdam UMC, University of Amsterdam, Amsterdam, The Netherlands; 2 Department of Biomedical Engineering, Erasmus MC, Rotterdam, the Netherlands; 3 Departments of Bioengineering, Imperial College London, London, UK; 4 Department of Radiology and Nuclear Medicine, Erasmus MC, Rotterdam, the Netherlands; 5 Department of Epidemiology, Erasmus MC, Rotterdam, the Netherlands

**Keywords:** Atherosclerosis, Carotid artery, Mechanical stress, Wall thickness

## Abstract

**Background:**

To explore the possibility of creating three-dimensional (3D) estimation models for patient-specific wall thickness (WT) maps using patient-specific and cohort-averaged WT, wall shear stress (WSS), and vessel diameter maps in asymptomatic atherosclerotic carotid bifurcations.

**Methods:**

Twenty subjects (aged 75 ± 6 years [mean ± standard deviation], eight women) underwent a 1.5-T MRI examination. Non-gated 3D phase-contrast gradient-echo images and proton density-weighted echo-planar images were retrospectively assessed for WSS, diameter estimation, and WT measurements. Spearman’s ρ and scatter plots were used to determine correlations between individual WT, WSS, and diameter maps. A bootstrapping technique was used to determine correlations between 3D cohort-averaged WT, WSS, and diameter maps. Linear regression between the cohort-averaged WT, WSS, and diameter maps was used to predict individual 3D WT.

**Results:**

Spearman’s ρ averaged over the subjects was − 0.24 ± 0.18 (*p* < 0.001) and 0.07 ± 0.28 (*p* = 0.413) for WT versus WSS and for WT versus diameter relations, respectively. Cohort-averaged ρ, averaged over 1000 bootstraps, was − 0.56 (95% confidence interval [− 0.74,− 0.38]) for WT versus WSS and 0.23 (95% confidence interval [− 0.06, 0.52]) for WT versus diameter. Scatter plots did not reveal relationships between individual WT and WSS or between WT and diameter data. Linear relationships between these parameters became apparent after averaging over the cohort. Spearman’s ρ between the original and predicted WT maps was 0.21 ± 0.22 (*p* < 0.001).

**Conclusions:**

With a combination of bootstrapping and cohort-averaging methods, 3D WT maps can be predicted from the individual 3D WSS and diameter maps. The methodology may help to elucidate pathological processes involving WSS in carotid atherosclerosis.

**Electronic supplementary material:**

The online version of this article (10.1186/s41747-018-0058-1) contains supplementary material, which is available to authorized users.

## Key points

- 3D cohort-averaging elucidates spatial correlations between wall shear stress and wall thickness in the carotid bifurcation
- Regression parameters based on cohort-averaged maps can predict individual wall thickness in 3D
- Individual estimation models could help tailor early management of atherosclerosis

## Background

Ischemic stroke occurs when an artery becomes occluded, leading to death of brain tissue [1]. Such an occlusion commonly occurs after rupture of an atherosclerotic lesion. Atherosclerotic plaques predominantly form at predilection sites with disturbed blood flow, such as arterial bifurcations [2]. The carotid bifurcation, particularly the lateral wall of the internal carotid artery (ICA), is a region prone to early atherosclerotic lesion formation, characterized by vessel wall thickening.

Wall shear stress (WSS) is the tangential friction force exerted by blood flow on the endothelial cells and is a known mediator of vessel calibre and endothelial function [3]. It is implicated in the development of atherosclerosis. For example, WSS is low at the lateral wall of the ICA [4], which may explain the predilection for disease in this region. It is important to measure WSS for the investigation of atherosclerotic processes [5]. If the relationship between WSS and vessel wall thickening is clarified further, the wall thickness (WT) can potentially be predicted from WSS patterns. Such an estimation has the potential to be clinically relevant in the management of atherosclerotic disease. For example, patients with abnormally low WSS can be monitored more closely to evaluate thickening of the wall and further disease progression and eventually facilitate timely treatment.

Three-dimensional (3D) phase-contrast magnetic resonance imaging (MRI) with velocity encoding in the three principal directions permits the estimation of WSS in the carotid bifurcation in vivo [6]. Advances in algorithms to estimate WSS from phase-contrast MRI data now allow the acquisition of full 3D WSS profiles along the entire bifurcation [7–10]. Recently, a method was introduced to create 3D maps that represent the average WSS of a certain cohort [11]. This technique enables local statistical comparisons to elucidate pathological processes involving 3D WSS [12].

WT may be affected by factors other than atherosclerosis; in particular, it is known that healthy arterial walls are dimensioned according to the law of Laplace [13], which states that tension in the wall increases with vessel diameter, resulting in increased WT [14]. Consequently, WT may correlate with WSS because both are independently determined by vessel diameter. For this reason, vessel diameter was also characterized and investigated. The relationships between variables were investigated by statistical comparison of maps, taking spatial autocorrelation into account [15].

The aim of this study was to explore the possibility of creating 3D estimation models for patient-specific WT maps using cohort-averaged WT, WSS and vessel diameter maps in asymptomatic atherosclerotic carotid bifurcations. We hypothesize that the methodology can predict 3D WT maps that are similar to the original WT maps.

## Methods

### Subject enrolment

Twenty subjects aged 75 ± 6 years (mean ± standard deviation, range 55–82 years, eight women) having asymptomatic plaques in the left carotid artery were retrospectively selected from the Rotterdam Study. The Rotterdam study is a population-based study of subjects aged ≥ 45 years investigating determinants of disease among the elderly [16]. The local Medical Ethics Committee approved the study and all participants provided informed consent. The study was performed in accordance with the declaration of Helsinki. All subjects underwent a two-dimensional ultrasound examination of the left carotid artery during which it was determined that WT was ≥ 2.5 mm at least at one location in the carotid artery [17].

### MRI protocol

The carotid MRI examination, performed with a 1.5-T scanner (Signa Excite II; GE Healthcare, Milwaukee, WI, USA), included non-gated, time-averaged 3D phase-contrast imaging (field of view 180 × 180 mm^2^; 40 slices; spatial resolution 0.7 × 0.7 × 1 mm^3^, echo time 4.3 ms; repetition time 13 ms, velocity encoding 60 cm/s) and proton density-weighted echo planar imaging (field of view 130 × 70 mm^2^; 51 slices; spatial resolution 0.5 × 0.5 × 1.2 mm^3^; echo time 24.3 ms; repetition time 12,000 ms). The phase-contrast images were corrected for background phase offsets by subtraction of the velocity in static tissue (the sternocleidomastoid muscle).

### MRI post-processing

The vessel lumen and wall were manually segmented in the proton density-weighted echo-planar images using ITK-snap (version 3.2, www.itksnap.org) [18]. The wall was converted to a mesh to enable 3D WT calculations. WT was quantified as the shortest distance between a mesh point and the outer wall (that included the adventitia). Rigid co-registration of the proton density-weighted echo-planar images and 3D flow images was performed in Elastix [19] to ensure that identical lumen segmentation was used for calculation of 3D WSS and 3D diameter.

WSS was quantified from the 3D flow images as previously described [8]. To derive the velocity gradient at the wall, smoothing splines were fitted through the rotated x- and y-velocity values using three equidistant points along the inward normal. The length of the inward normal used for WSS estimation was the radius of the vessel. All maps consisted of the common carotid artery (CCA), the bifurcation, the proximal ICA and the proximal external carotid artery.

To calculate the vessel diameter in 3D, the vascular modelling toolkit VMTK [20] was used to: 1) calculate the centreline of the lumen; 2) calculate the inward distance from the luminal surface to the centreline; and 3) multiply the distance to the centreline (i.e. the radius) by a factor of 2 to yield the diameter on each point of the vessel wall.

### Cohort-averaged maps

Cohort-averaged maps for WSS, WT and diameter were created by: 1) creation of a shared geometry; 2) interpolation of the individual WSS, WT and diameter values to the shared geometry; and 3) averaging of WSS, WT and diameter on the shared geometry over the cohort [11].

### Statistical analysis

Spearman’s rank correlation coefficient ρ was determined for the correlations between subject-specific WSS and WT, WT and diameter as well as WSS and diameter, using all pixels. A Fisher z-transformation was applied to normalize ρ, enabling a Student’s *t*-test to determine if the z-values averaged across subjects were significantly different from 0. *p* values lower than 0.05 were considered significant. Linear regression was performed as well and R^2^ reported. A linearity check was additionally performed.

For the assessment of *ρ* between the cohort-averaged maps using all pixels, the bootstrapping method was used [15]: the 20 individual WT, WSS and diameter maps were randomly sampled (with replacement) followed by 3D averaging. Such a cohort-averaged map thus contained the map of the same subject multiple times. Random sampling and averaging was repeated 1000 times (i.e. a bootstrapping size of 1000 was used). ρ was determined for each combination of randomly sampled and averaged WT and WSS maps (*ρ*_*WSS* − *WT*_), for each combination of WT and diameter maps (*ρ*_*WT* − *D*_) and for each combination of WSS and diameter maps (*ρ*_*WSS* − *D*_), using all pixels. For these combinations, ρ was significant when the 95% confidence interval (CI) of the ρ values did not contain 0.

### Linear regression for WT estimation based on individual maps

For each subject *i*, linear regression was performed for all pixels using Eq. 1:

1 $$ {WT}_i={\beta_0}_i+{\beta_1}_i\ast {WSS}_i+{\beta_2}_i\ast {D}_i+{\beta_3}_i\ast {WSS}_i\ast {D}_i+{\varepsilon}_i $$

where *WT*_*i*_ is the 3D WT map, *WSS*_*i*_ is the 3D WSS map and *D*_*i*_ is the 3D diameter map. *β*_0*i*_ is the intercept and *β*_1*i*_, *β*_2*i*_ and *β*_3*i*_ are the regression coefficients; *ε*_*i*_ is the residual.

The regression coefficients were used to compute the 3D WT map for each subject. The inclusion of *β*_2*i*_ ∗ *D*_*i*_ follows from the law of Laplace. The inclusion of *β*_3*i*_ ∗ *WSS*_*i*_ ∗ *D*_*i*_ follows from the vessel calibre regulatory mechanisms of WSS [3, 4].

### Linear regression for WT estimation based on cohort-averaged maps

Two linear regression analyses were performed for estimation of patient-specific 3D WT maps. First, linear regression was performed for all 1000 bootstrapped cohort-averaged WT, WSS and diameter maps using the equation for all pixels:

2 $$ {\overline{WT}}_b={\beta_0}_b+{\beta_1}_b\ast {\overline{WSS}}_b+{\beta_2}_b\ast {\overline{D}}_b+{\beta_3}_b\ast {\overline{WSS}}_b\ast {\overline{D}}_b+{\varepsilon}_b $$

where ${\overline{WT}}_b$ is one bootstrap *b* of the cohort-averaged 3D WT map, ${\overline{WSS}}_b$ is one bootstrap *b* of the cohort-averaged 3D WSS map and ${\overline{D}}_b$ is one bootstrap *b* of the cohort-averaged 3D diameter map. *β*_0*b*_ is the intercept and *β*_1*b*_, *β*_2*b*_ and *β*_3*b*_ are the regression coefficients per bootstrap. *ε*_*b*_ is the residual per bootstrap. Thus, 1000 *β*_0_, *β*_1_, *β*_2_ and *β*_3_ coefficients are obtained by solving this regression equation. The mean and 95% CI of the 1000 *β*_0_, *β*_1_, *β*_2_ and *β*_3_ are reported.

Second, the average of the 1000 *β*_*i*_ ($\overline{\beta_0}$, $\overline{\beta_1}$ and $\overline{\beta_2}$) are used to predict the individual 3D WT maps from the individual 3D WSS and diameter maps for all pixels.

**Fig. 1:**
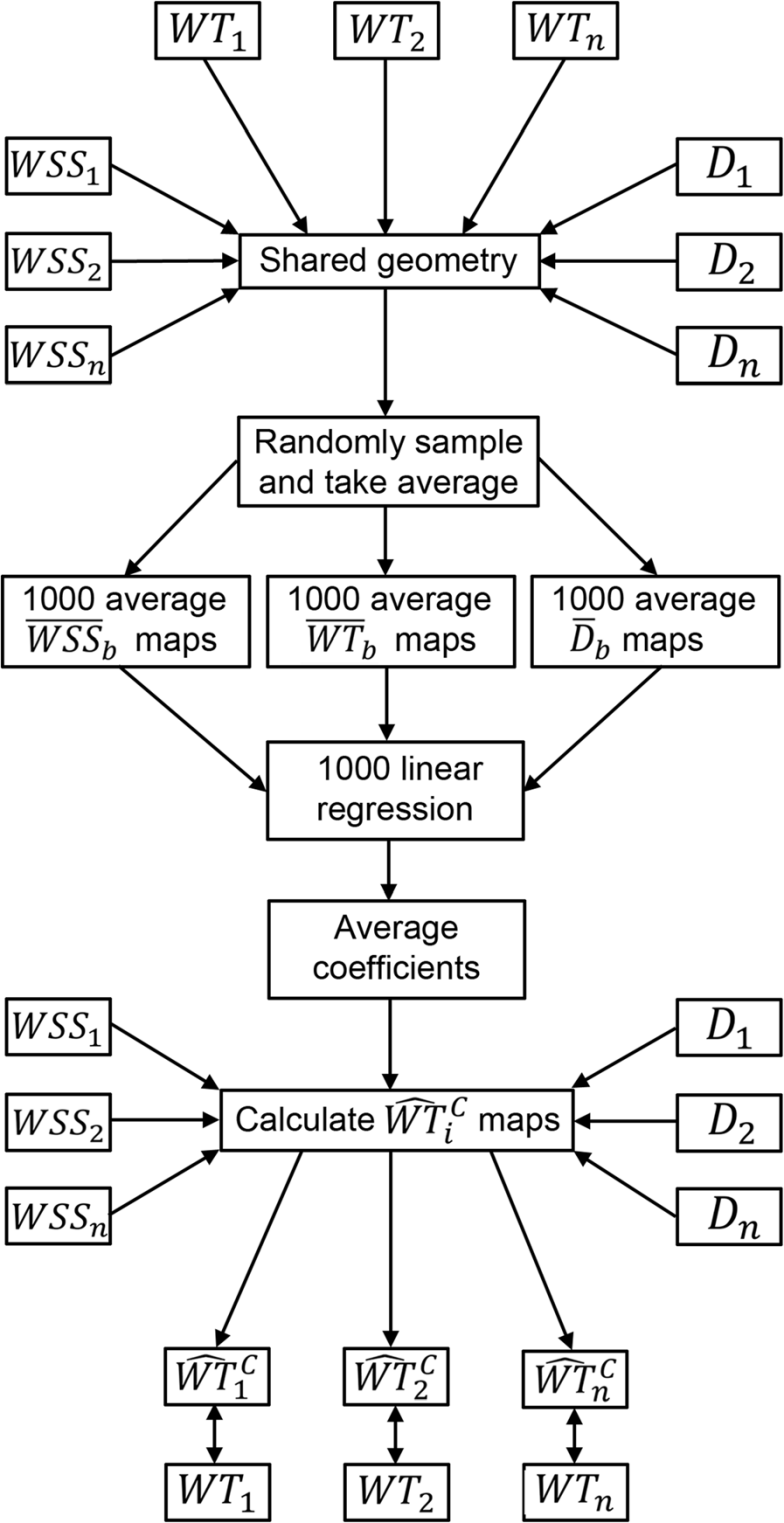
Schematic overview of the workflow for predicting wall thickness maps from cohort-averaged wall shear stress and diameter maps. *WT* wall thickness, *WSS* wall shear stress, *D* diameter, ${\widehat{WT}}_i^C$ predicted wall thickness

An overview of the workflow for predicting WT maps from cohort-averaged WSS and diameter maps is given in Fig. 1. To investigate the influence of WSS, diameter and the interaction factor WSS*D separately, the two-step linear regression process is repeated by including only WSS, diameter or the interaction term in Eqs. 1 to 4.

### Comparison between estimated and measured WT maps

For the individual and cohort-averaged estimation results, Spearman’s ρ was determined using all pixels per subject to quantify the agreement between the estimated and the original WT map. Bland-Altman analysis was performed and the mean difference and limits of agreement averaged over the subjects reported. Furthermore, maximum WT, defined as the average of the top 5% of all values, was quantified for the original and predicted WT maps.

## Results

### Individual analysis

**Fig. 2:**
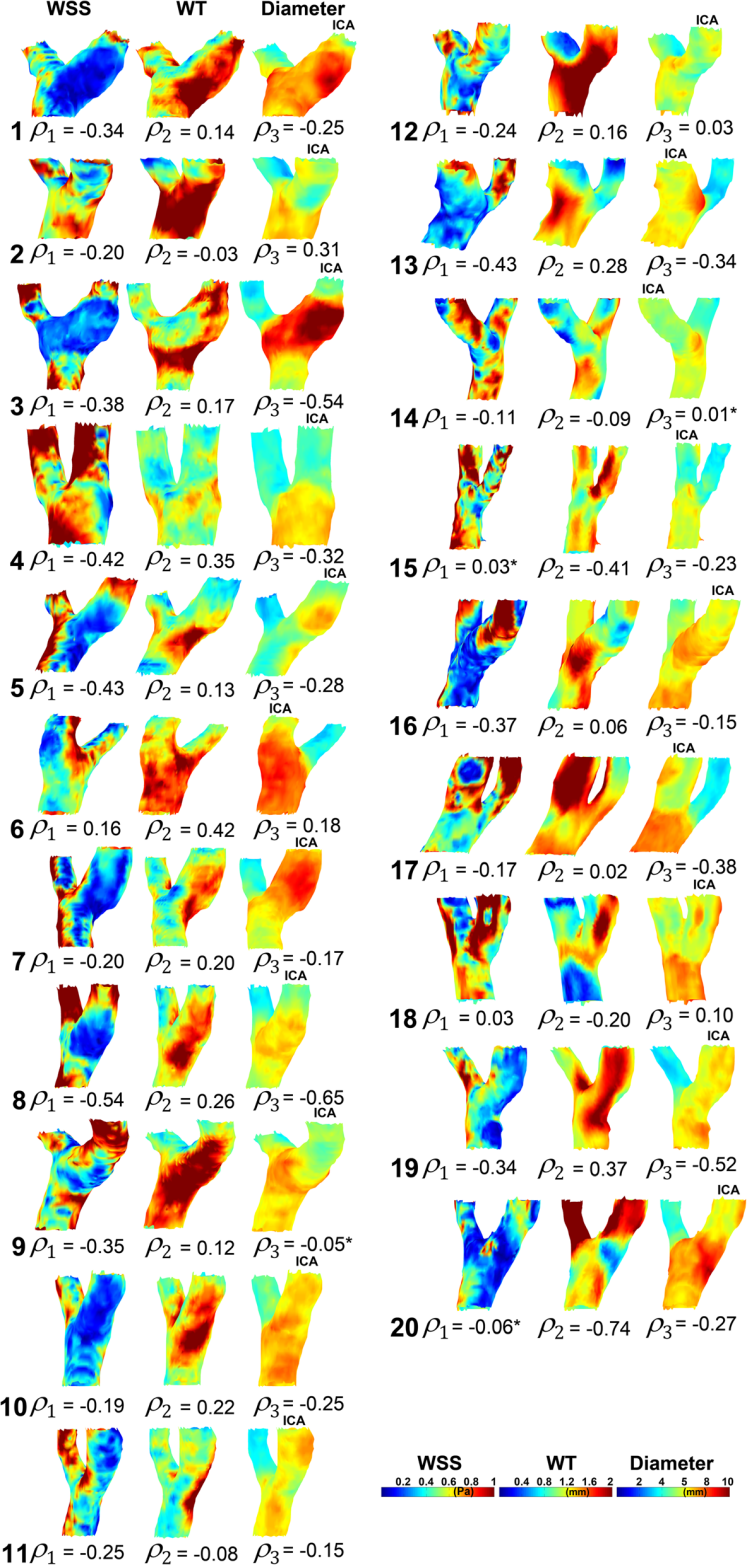
Wall shear stress (*WSS*), wall thickness (*WT*) and diameter maps of the left carotid bifurcation for all subjects. The internal carotid artery (*ICA*) is indicated. Spearman’s ρ for WSS–WT (ρ_1_), WT–diameter (ρ_2_) and WSS–diameter (ρ_3_) is given. Asterisks indicate where the linear model was inappropriate

Figure 2 displays the 3D WSS, WT and diameter maps for all subjects. Spearman’s ρ for the WSS and WT relationship and for the WT and diameter relationship is also shown. *ρ*_*WT* − *WSS*_ averaged over all subjects was − 0.24 ± 0.18 (*p* < 0.001). *ρ*_*WT* − *D*_ averaged over the subjects was 0.07 ± 0.28 (*p* = 0.413). *ρ*_*WSS* − *D*_ averaged over all subjects was − 0.20 ± 0.24 (*p* = 0.002).

Additional file 1: Figure S1a and b display, respectively, the scatter plots and linear regression lines for every individual WT–WSS relationship. Mean R^2^ was 0.08 ± 0.07. Additional file 1: Figure S1c and d display the scatter plots and linear regression lines for every individual WT–diameter relationship. Mean R^2^ was 0.09 ± 0.15. Additional file 1: Figure S1e, f display the scatter plots and linear regression lines for every individual WSS–diameter relationship. Mean R^2^ was 0.11 ± 0.12. For most subjects, a negative and positive relationship between WSS and WT and between diameter and WT, respectively, was found. For most subjects, a negative relationship was found between WSS and diameter. This is also displayed in Fig. 2.

### Cohort-averaged analysis

**Fig. 3:**
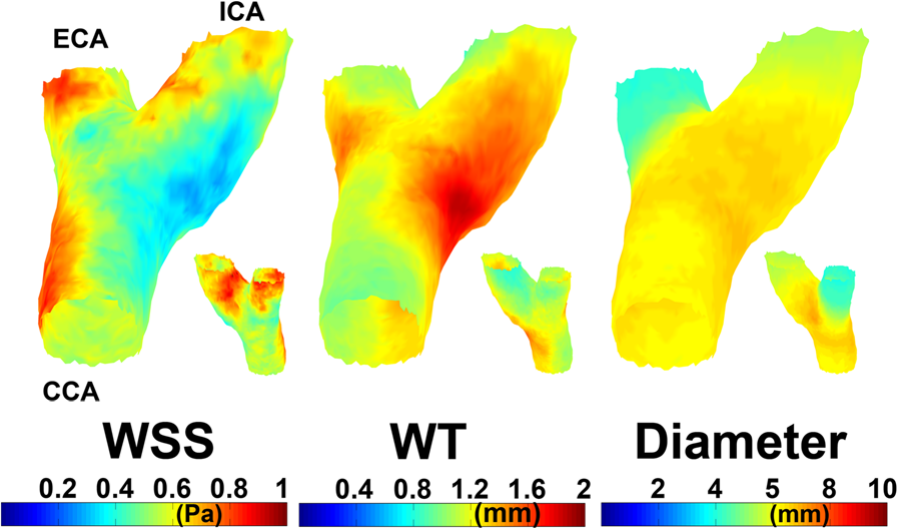
The mean map of 1000 bootstraps of the left carotid bifurcation for wall shear stress (*WSS*), wall thickness (*WT*) and vessel diameter. Insets show the 180° reversed view

The means of 1000 bootstrapped cohort-averaged 3D WT, WSS and diameter maps are shown in Fig. 3. Regions of low WSS showed good co-localization with regions of high WT. The mean [95% CI] of ρ for the 1000 WT-WSS relationship was − 0.56 [− 0.74, − 0.38]. Regions of large diameter also showed correspondence with regions of high WT; Spearman’s ρ was 0.23 [− 0.06, 0.52]; however, this 95% CI contained 0, thus implying lack of significance. Regions of low WSS showed good co-localization with regions of large diameter as well (Spearman’s *ρ* − 0.32 [− 0.51, − 0.14]).

Scatter plots with the linear regression line are displayed for the relationship between the mean of the 1000 bootstraps for WSS and WT, for diameter and WT and for WSS and diameter are shown in Additional file 1: Figure S2a, b, and c, respectively. R^2^ for these regression curves was 0.50, 0.06, and 0.12, respectively. The linear model was appropriate for all three regressions. Compared to Additional file 1: Figure S1, the variance in the data is small for all relationships (the same axis ranges have been used to facilitate comparison.)

### Predicted WT maps based on the individual maps

**Fig. 4:**
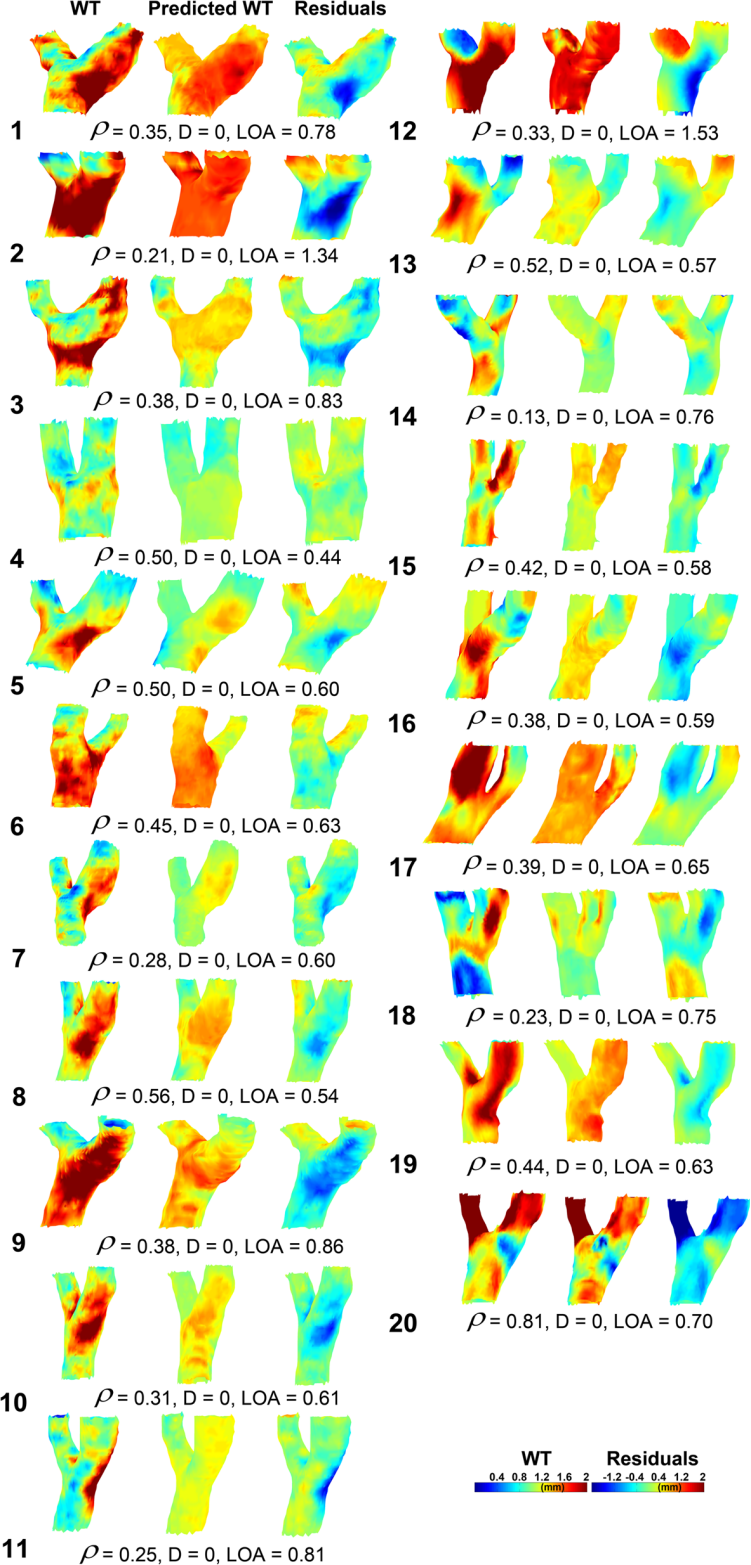
The predicted WT maps (*center column*) based on linear regression of the individual WT, WSS and diameter maps compared to the original WT maps (*left column*). The *right column* shows the 3D residual maps. Spearman’s ρ is given. *D* and *LOA* represent the difference and limits of agreement from the Bland-Altman analysis

Figure 4 displays the measured 3D WT maps and the predicted 3D WT maps for all subjects, created with the individual regression coefficients. The mean difference and limits of agreement were 0.00 ± 0.00 and 0.74 ± 0.26 mm, respectively. In Table 1, *ρ* between the original and predicted WT and the maximum WT is given for the total model, for WSS only, diameter only and the interaction term WSS*D. The total model predicted WT best.

**Table 1:** Spearman’s ρ and maximum WT for the agreement between the predicted WT maps and the original WT maps based the individual maps on the cohort-averaged maps

Prediction model	Quantification method	Individual maps	Cohort-averaged maps
Total model	Spearman ρ	0.39 ± 0.15 *p* < 0.001	0.21 ± 0.22 *p* < 0.001
	Maximum WT (mm)	1.7 ± 0.4	2.1 ± 0.3
WSS only	Spearman ρ	0.25 ± 0.16 *p* < 0.001	0.24 ± 0.18 *p* < 0.001
	Maximum WT (mm)	1.4 ± 0.2	1.7 ± 0.0
Diameter only	Spearman ρ	0.22 ± 0.18 *p* < 0.001	0.07 ± 0.28 *p* = 0.413
	Maximum WT (mm)	1.5 ± 0.4	1.4 ± 0.1
Interaction term: WSS*D only	Spearman ρ	0.25 ± 0.14 *p* < 0.001	0.22 ± 0.18 *p* < 0.001
	Maximum WT (mm)	1.4 ± 0.3	1.6 ± 0.0

All *p* values calculated as Student’s *t*-test after the Fisher z-transformation to determine significant difference with 0. *p* values lower than 0.05 were considered as significant. *WT* wall thickness, *WSS* wall shear stress, *D* diameter

### Predicted WT maps based on the cohort-averaged maps

**Fig. 5:**
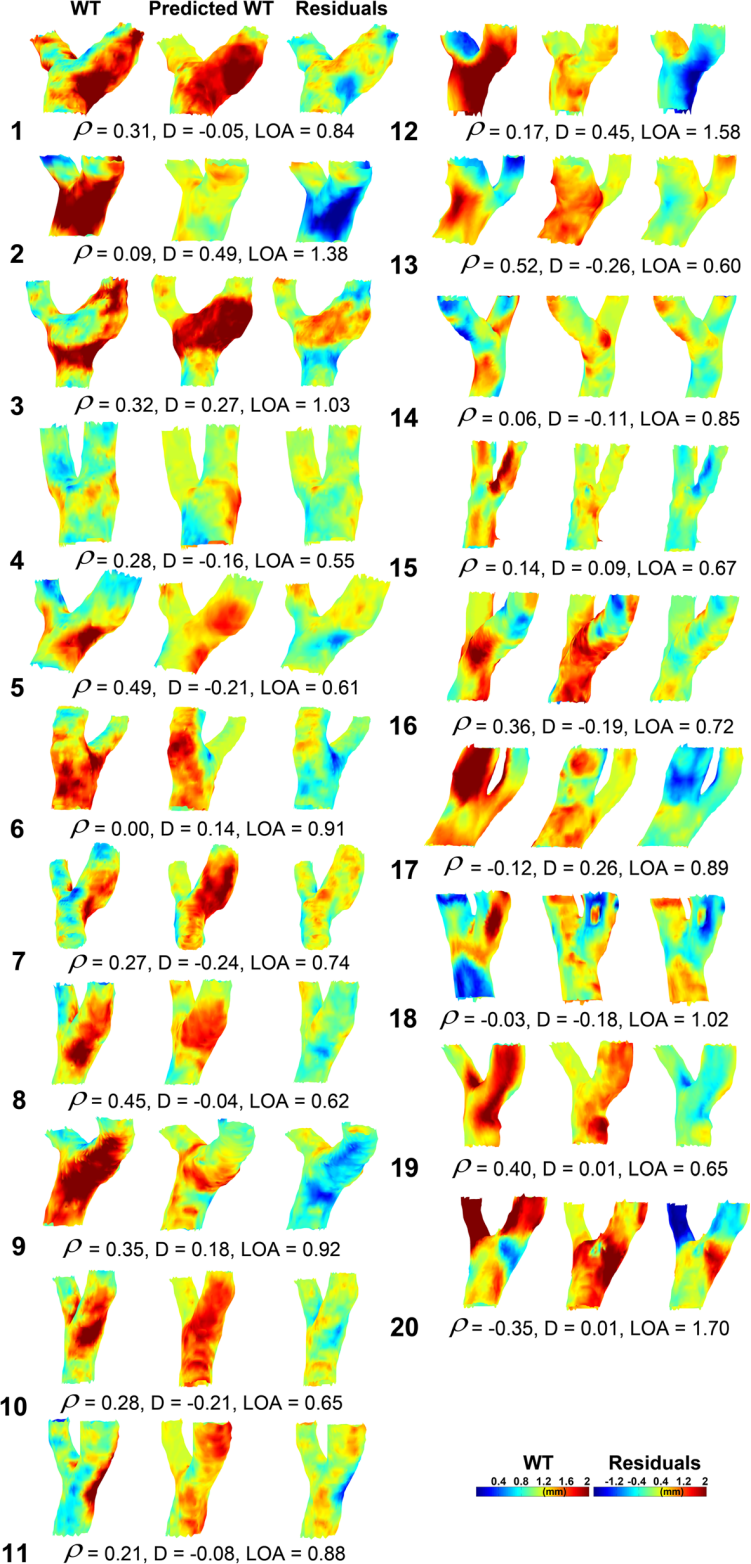
The predicted WT maps (*center column*) based on linear regression of the cohort-averaged WT, WSS and diameter maps compared to the original WT maps (*left column*). The *right column* shows the 3D residual maps. Spearman’s ρ is given. *D* and *LOA* represent the difference and limits of agreement from the Bland-Altman analysis

Figure 5 displays the measured 3D WT maps and the predicted WT maps for all subjects, created with the cohort-averaged regression coefficients averaged over all bootstraps. Three subjects had a negative ρ. For these subjects the estimation of WT was poor. The mean difference and limits of agreement were − 0.02 ± 0.23 and 0.89 ± 0.32 mm, respectively.

In Table 1, *ρ* between the original and predicted WT and the maximum WT is given for the total model, for WSS only, diameter only and the interaction term WSS*D. The WSS-only model predicted WT best in terms of ρ, whereas the total model predicted WT best based on maximum WT. Note that maximum WT for the total model based on the cohort-averaged maps was higher than estimation based on the individual maps. This is also apparent from Figs. 4 and 5.

## Discussion

This study provides a novel framework for investigating the relationship in a 3D manner between WSS, WT and vessel diameter. The work was based on advanced cohort averaging and statistical methods of data from the carotid bifurcation of patients with asymptomatic atherosclerosis, and it took account of spatial autocorrelation [21].

The main findings of this study were fourfold: 1) creating cohort-average maps of WSS, WT and diameter is feasible in the carotid bifurcation; 2) the relationship between WT and WSS was significant on an individual level and for the averaged maps; 3) WT did not correlate with diameter; 4) cohort-averaged WT and WSS maps can partially predict individual WT maps based on individual WSS and diameter maps.

Previous studies have also found correlations between WSS and WT in the CCA [22, 23]. Carallo et al. [14] compared WT with WSS derived from ultrasound measurements and with wall tension (blood pressure * diameter). A multiple regression analysis including WSS and wall tension as independent variables and WT as the dependent variable showed that WSS was the only independent variable and strongly associated with WT, whereas wall tension correlated only weakly with WT. In our study, blood pressure values were not available. Nonetheless, this finding supports our result that diameter had a smaller effect than WSS on WT. Indeed, our data support the notion of Carallo et al. that the relationship between wall tension and early atherosclerotic lesions is mediated by low WSS.

Steinman et al. [24] recognized that 3D maps of WSS, including the bifurcation, are helpful to investigate the aetiology and progression of carotid atherosclerosis. They performed MRI-based computational fluid dynamics calculation and found a qualitative agreement between low WSS and increased WT. They showed a scatter plot of WSS against WT in an individual with early, asymptomatic carotid artery disease. This graph looked very similar to our scatter plots. They concluded that no obvious relationship is apparent from such scatter plots. We demonstrated that such a relationship will become more evident when a scatter plot is created from cohort-averaged WSS and WT data. Thus, the correlation coefficients between cohort-averaged WT and WSS are suitable for estimating individual WT maps. Note that the proposed methodology is perfectly suitable for use on computational fluid dynamics data as well.

Augst et al. [25] used ultrasound for WT measurements and they performed computational fluid dynamics calculation to derive WSS in the CCA and in the carotid bifurcation. They found a significant negative correlation between WSS and WT in the CCA, but, contrary to our findings, not at the level of the carotid bifurcation. A possible explanation is the inclusion of 14 healthy controls, with low intima-media thickness (0.4–0.8 mm), whereas we included subjects with plaque and a WT range of 0.5–3 mm.

A limitation of our study is the absence of the temporal dimension in the phase-contrast MRI measurements. The consensus hemodynamic theory for explaining the localization of atherosclerosis combines low WSS and a high oscillatory shear index (OSI) [26]. There is evidence in mice that low WSS produces vulnerable plaques, whereas high OSI induces stable lesions [27]. Markl et al. [6] measured OSI in two-dimensional slices placed perpendicular to the carotid using four-dimensional phase-contrast MRI and showed that a fraction of the carotid bulb was exposed to high OSI. Thus, with inclusion of cardiac gating in the phase-contrast MRI exam and in combination with novel techniques to produce motion-resolved segmentation [28], both four-dimensional WSS and 3D OSI maps can be derived in the future.

Another limitation is that we did not have a sufficient number of subjects to create a “train” and “test” cohort. Here we aimed to demonstrate the feasibility of the technique for creating predictive models. In future studies we will test the methodology more rigorously.

In this initial study, no follow-up data were available. Longitudinal monitoring of combined carotid WSS and OSI may provide more insight into the initiation, growth, and rupture of carotid plaques. We plan to create a framework that is able to predict carotid WT based on WSS estimations and other risk factors in daily clinical practice, with the aims of detecting carotid atherosclerosis early and providing disease management tailored to the individual patient.

If consensus is reached in the community about wall thickening occurring in regions of low WSS, we may be able to detect atherosclerosis at a very early stage, even before wall thickening occurs. Early detection will facilitate patient management and could reduce healthcare costs. For example, with the combined assessment of WSS and other risk factors such as age, smoking and cholesterol status, the patient’s 3D WT map could be predicted and compared to other WT maps created using similar subjects, with known clinical outcome. Thus, a risk profile for the patient could be created with the techniques described in this paper. A risk profile would allow for early initiation of medical therapies, dietary advice or exercise strategies. With more patient characteristics available, the prediction model can be advanced into more complex statistical models [29].

In conclusion, 3D statistical methods, incorporating corrections for spatial autocorrelation, were applied to predict 3D individual WT maps using cohort-averaged 3D WSS and diameter maps. With the inclusion and careful categorization of more subjects, the predictive value of WSS for wall thickening is likely to improve, enabling early detection of atherosclerosis with the purpose of better patient management. The methodology presented may help to elucidate the pathological process between hemodynamic forces and wall thickening in carotid atherosclerosis and ultimately prove useful for the diagnosis of plaque initiation and monitoring of disease progression.

## Additional file

Additional file 1: **Figure S1.** **a** Scatter and **b** linear regression plots for all subjects for the relationship between 3D WSS and 3D WT. **c** Scatter and **d** linear regression plots for all subjects for the relationship between 3D diameter and 3D WT. **e** Scatter and **f** linear regression plots for all subjects for the relationship between 3D WSS and 3D diameter. **Figure S2.** **a** Scatter plot with the linear regression line for the bootstrap-averaged 3D WSS and 3D WT map. **b** Scatter plot with the linear regression line for the bootstrap-averaged 3D WT and 3D diameter map. **c** Scatter plot with the linear regression line for the bootstrap-averaged 3D WSS and 3D diameter map. The colours indicate the density of the data points. (DOCX 808 kb)

## Abbreviations

3D: Three-dimensional
CCA: Common carotid artery
CI: Confidence interval
D: Diameter (*in formulas*)
ICA: Internal carotid artery
OSI: Oscillatory shear index
WSS: Wall shear stress
WT: Wall thickness
