# Supplementary material for: Spatial correlations between MRI-derived wall shear stress and vessel wall thickness in the carotid bifurcation
Source: Eur Radiol Exp. 2018 Oct 10;2:27. doi: 10.1186/s41747-018-0058-1 (PMC6177500; doi:10.1186/s41747-018-0058-1)
Supplement: Supplementary file 1 — Figure S1. a Scatter and b linear regression plots for all subjects for the relationship between 3D WSS and 3D WT. c Scatter and d linear regression plots for all subjects for the relationship between 3D diameter and 3D WT. e Scatter and f linear regression plots for all subjects for the relationship between 3D WSS and 3D diameter. Figure S2. a Scatter plot with the linear regression line for the bootstrap-averaged 3D WSS and 3D WT map. b Scatter plot with the linear regression line for the bootstrap-averaged 3D WT and 3D diameter map. c Scatter plot with the linear regression line for the bootstrap-averaged 3D WSS and 3D diameter map. The colours indicate the density of the data points. (DOCX 808 kb) [file 41747_2018_58_MOESM1_ESM.docx]

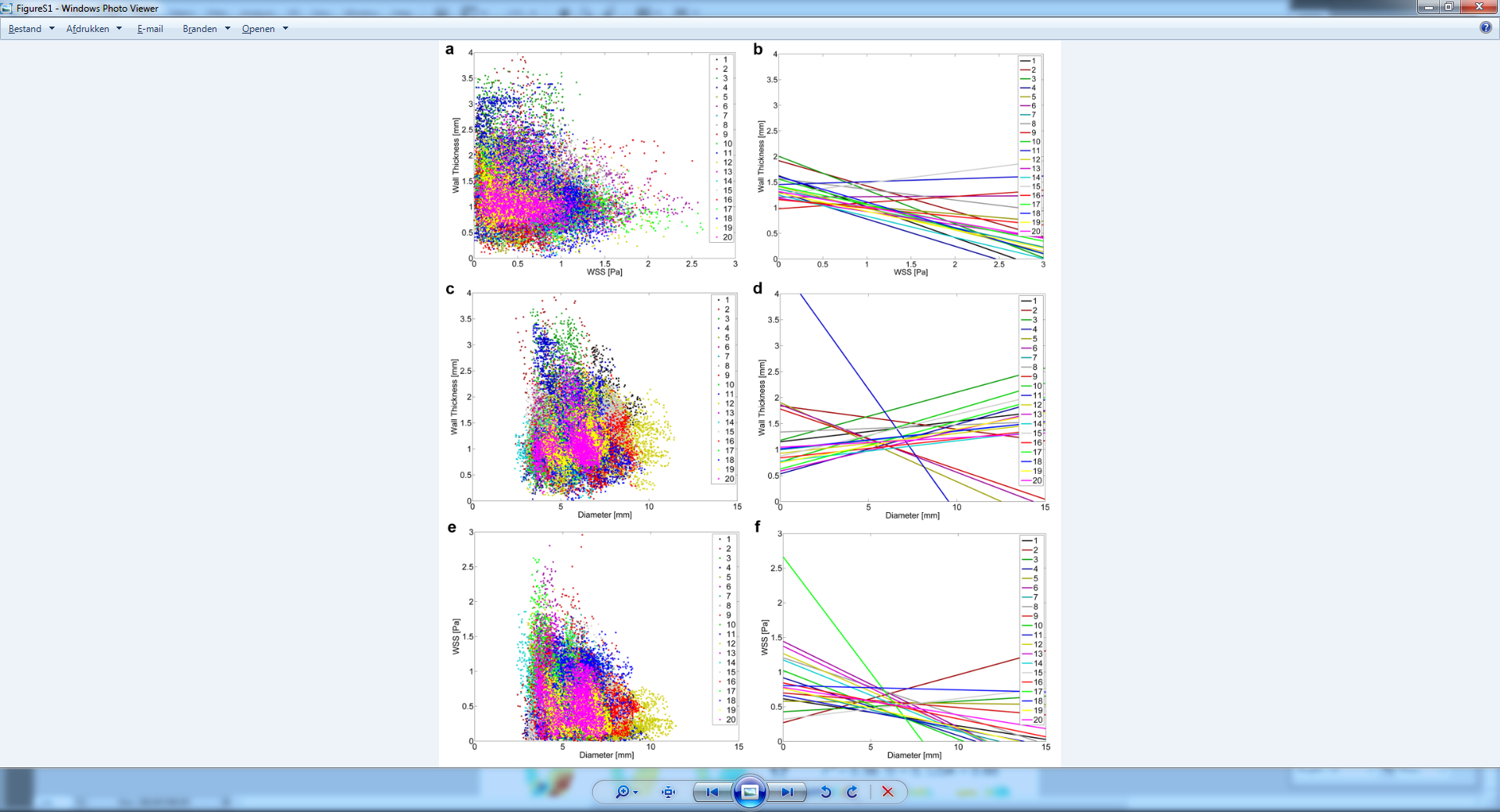


***Figure S1****. a) Scatter and b) linear regression plots for all subjects for the relationship between 3D WSS and 3D WT. c) Scatter and d) linear regression plots for all subjects for the relationship between 3D diameter and 3D WT. e) Scatter and f) linear regression plots for all subjects for the relationship between 3D WSS and 3D diameter.*


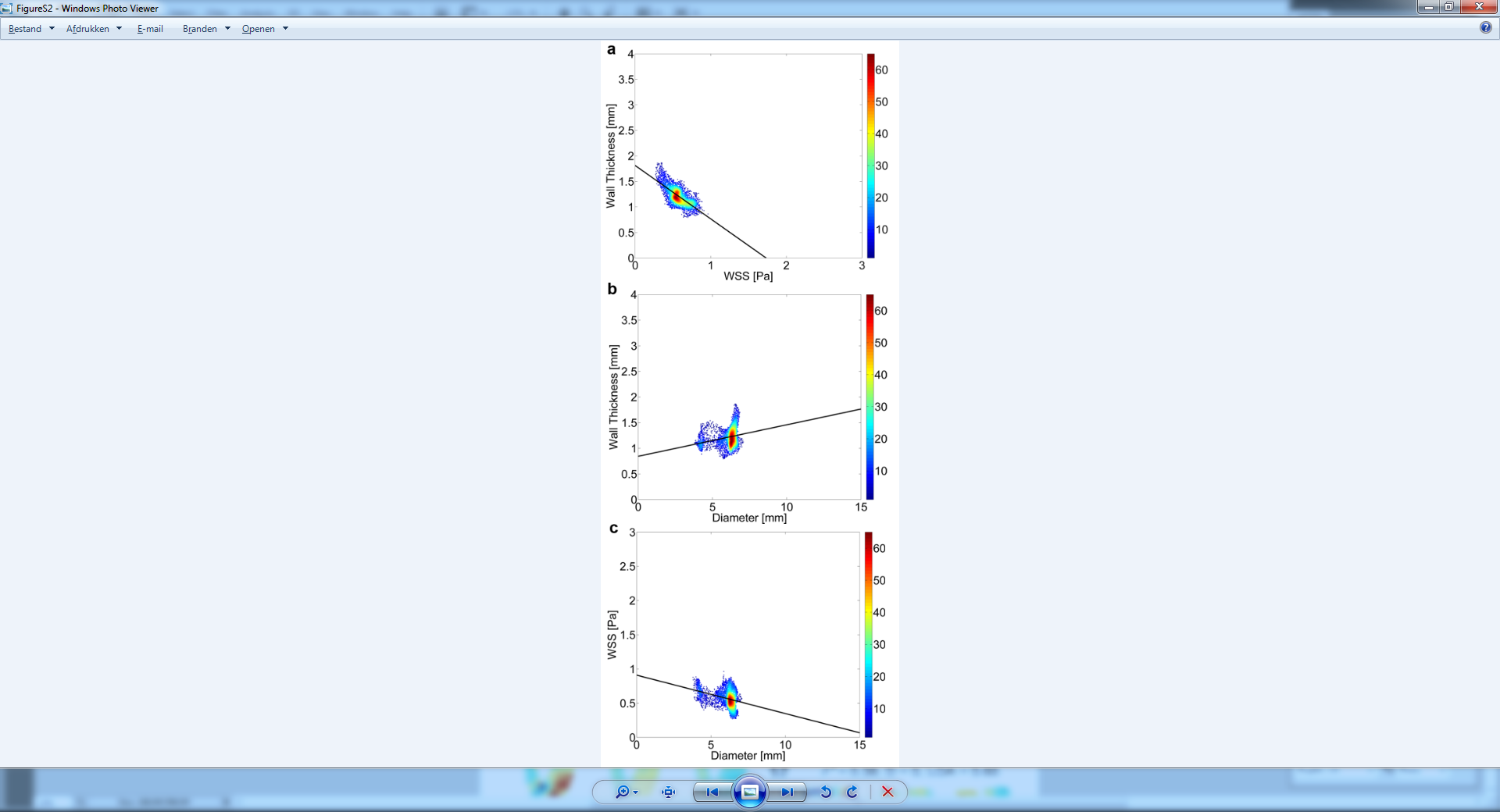


***Figure S2****. a) Scatter plot with the linear regression line for the bootstrap-averaged 3D WSS and 3D WT map. b) Scatter plot with the linear regression line for the bootstrap-averaged 3D WT and 3D diameter map. c) Scatter plot with the linear regression line for the bootstrap-averaged 3D WSS and 3D diameter map. The colors indicate the density of the data points.*
